# Long Term Memory Outcome of Repetitive, Low-Level Dietary Exposure to Domoic Acid in Native Americans

**DOI:** 10.3390/ijerph18083955

**Published:** 2021-04-09

**Authors:** Lynn M. Grattan, Laura Kaddis, J. Kate Tracy, John Glenn Morris

**Affiliations:** 1 Department of Neurology, University of Maryland School of Medicine, Baltimore, MD 21201, USA; 2 Department of Otorhinolaryngology, University of Maryland School of Medicine, Baltimore, MD 21201, USA; LKaddis@som.umaryland.edu; 3 Department of Epidemiology and Public Health, University of Maryland School of Medicine, Baltimore, MD 21201, USA; KTracy@som.umaryland.edu; 4 Department of Medicine, College of Medicine, Emergency Pathogens Institute, University of Florida, Gainesville, FL 32610, USA; JGMorris@epi.ufl.edu

**Keywords:** domoic acid and human health, amnesic shellfish poisoning, harmful algal blooms, *Pseudo-nitzchia*, shellfish safety

## Abstract

Domoic acid (DA) is a marine-based neurotoxin that, if ingested via tainted shellfish, is associated with *Amnesic Shellfish Poisoning* (ASP). These acute effects of elevated DA exposure in humans have been well described. In contrast, the long-term impacts of lower level, repetitive, presumably safe doses of DA (less than 20 ppm) are minimally known. Since Native Americans (NA) residing in coastal communities of the Pacific NW United States are particularly vulnerable to DA exposure, this study focuses on the long-term, 8-year memory outcome associated with their repeated dietary consumption of the neurotoxin. Measures of razor clam consumption, memory, clerical speed and accuracy, and depression were administered over eight years to 500 randomly selected adult NA men and women ages 18–64. Data were analyzed using GEE analyses taking into consideration the year of study, demographic factors, and instrumentation in examining the association between dietary exposure and outcomes. Findings indicated a significant but small decline in total recall memory within the context of otherwise stable clerical speed and accuracy and depression scores. There is reason to believe that a continuum of memory difficulties may be associated with DA exposure, rather than a unitary ASP syndrome.

## 1. Introduction

Harmful algal blooms have been increasing in frequency, intensity and duration for more than three decades in coastal regions around the world, contributing to an increase in public health concerns about fish and shellfish safety [1]. Domoic Acid (DA), a neurotoxin, is produced by harmful algal blooms that include toxic species of the diatom, *pseudo-nitzschia*. These blooms typically occur in temperate or subtropical ocean waters under specific, but not yet fully identified environmental conditions. These include ocean upwelling which brings the cells to the surface, and warm water anomalies that allow the algae to multiply [2,3]. Shellfish and other organisms feed on toxic pseudo-nitzschia, concentrate the toxin within them and through this process, DA enters the foodweb [4]. Over the past 30 years, persistent low levels of DA have been interspersed with dangerously high levels along the United States Pacific Coast, extending from northern Alaska to southern California [5,6]. When humans, marine mammals, or other wildlife consume the tainted shellfish, outbreaks of neurotoxicity occur [7,8,9,10,11,12,13,14,15,16]. In Washington State, Oregon, and California, DA was found to be responsible for numerous outbreaks of toxicity affecting fish, shellfish, shorebirds and sea lions [12,13,17,18] triggering a particular risk to coastal communities of the U.S. Pacific Northwest (PNW). DA and it precursor, toxic *Pseudo-nitzschia* has also been documented in other regions of the U.S. including Maine, Massachusetts, and Rhode Island, as well as off the coast of Argentina, Scotland, France, Ofunato Bay, Japan and continues to grow in its biogeographic distribution [19,20,21,22].

The potential risk of DA to human health was discovered in 1987, in Montreal, Canada [9,10,23]. Persons who ate affected mussels harvested from the Prince Edward Island Region suffered serious medical illnesses and, in four cases, death. Based on 107 people who met the criteria for the outbreak case definition, symptoms included vomiting, abdominal cramps, diarrhea, headache, seizures, and coma [9,10,23]. Several survivors were left with a permanent anterograde memory disorder, and autopsy results indicated significant cell death in the hippocampus, a cerebral region associated with memory [23]. Thus, the neurotoxic syndrome associated with DA exposure was named, *Amnesic Shellfish Poisoning* (ASP).

Analysis of mussel meal remnants indicated exposures within the range of 310 to 1280 ppm of DA [10]. Further analysis of these data led Wright et al. [12] to conclude that symptomatic individuals typically ingested a maximum dose of 200 to 300 mg of domoic acid. The patient with the fewest symptoms in the Canadian outbreak was reportedly exposed to 50 mg. This lowest minimally symptomatic dose was used to calculate the current regulatory limit in the United States and Canada of 20 ppm of DA in shellfish, taking into consideration a shellfish consumption rate of 200 g, and a safety factor of approximately 12 [24]. These action levels have been effective in preventing acute toxicity.

Of important contemporary concern is whether or not repetitive exposure to lower, presumably “safe” levels of DA over time can be harmful to human health. Studies of laboratory rats and mice identified problems with learning, memory, and spontaneous behavior associated with repeated, low-level exposure to DA [25,26,27,28]. In the natural environment, Cook and colleagues [11] identified spatial memory deficits in sea lions associated with repeated DA exposure over time. In this study, they found that older animals had more gliosis and inflammation in the area of the hippocampus, the cerebral structure associated with memory. The authors concluded that this reflected a DA toxicosis, which was distinguishable from the acute syndrome found after high-level exposures. In response to questions about the potential human health impacts of repetitive or cumulative sub-acute exposures to putatively “safe” levels of dietary DA exposure a cohort of 500 Native American (NA) adults residing on the NW Pacific coast. Early findings of this four-year cohort study [7] and others [29,30] found that repetitive or cumulative sub-acute exposures to putatively “safe” levels of DA in razor clams (*Siliqua patula*) in the US Pacific NW were at risk for memory decline. Specifically, people who consumed 15 or more razor clams per month demonstrated a mild decrement in performance (ASP in attenuated form). This occurred within the context of otherwise stable cognition. The effect size was low, thus an extended study was indicated. The purpose of this study was to examine the effects of repeated DA exposure over eight years upon memory in the NA adult cohort.

## 2. Materials and Methods

Study participants included 500 members of a Pacific NW NA cohort ages 18 years and older. The cohort represents NAs, randomly selected from the registries of three tribal nations who by virtue of access to coastal razor clam beaches, and traditional diets, regularly consume varying amounts of razor clams [31]. Participants were seen over 8 years for data collection.

Demographic and medical history were obtained using standardized interview procedures. Substance use, and razor clam consumption history were obtained using the brief Michigan Alcoholism Screen Test (BMAST) [32], Drug Abuse Screen Test (DAST) [33], and Shellfish Assessment Survey, a previously validated measure of razor clam exposure that assesses frequency, quantity and preparation method of the clams [34]. For razor clam exposure analysis, the yearly average for each participant was calculated, combining data from spring/summer and fall/winter seasons. Categories of razor clam consumption were: no clams consumed that year, low consumption (1–14 clams consumed per month), and high consumption (15+ clams consumed per month) based upon data from our previous study. Participants were able to move between different consumption categories during the study period. DA levels during the study period ranged from non-detectable to 14 ppm with most frequently occurring levels within the 3–5 ppm range.

The primary outcome of interest was memory. Clerical speed and accuracy, as well as mood, were assessed as secondary outcomes. The memory functions assessed were short delay recall (the number of words from a list recalled immediately after multiple presentations), long term recall (the number of words recalled 25 min after the final list presentation) and total recall (total of all words remembered throughout the task, including short and long term recall) using a word list learning and recall paradigm. Specifically, the California Verbal Learning Test—Second Edition (CVLT–II) [35] was used for years one through four, and the Hopkins Verbal Learning Test—Revised (HVLT-R) [36] was used during years seven and eight. The change in measures was in response to a practice effect (inflated scores due to taking the test multiple times), which we initially managed by using a different memory paradigm (years five and six), then returned to the alternate word lists of the HVLT. Both the HVLT and CVLT memory measures have been strongly correlated in a healthy adult sample [37]. The Digit Symbol Substitution subtest of the Weschler Adult Intelligence Scale—Third Edition [38] was used as a measure of clerical speed and accuracy. In this task, participants are presented with a key consisting of the numbers 1–9. Each number is paired to a unique, simple to draw symbol such as “V”, “+” or “>“. They are then presented with a random array of numbers 1–9 with a blank space below each number. The participant is asked to quickly scan the original answer key and fill in the blank space with the correct symbol for each corresponding number. The score is based upon how many correct symbols they place in 120 seconds. The Beck Depression Inventory (BDI) [39] was used to assess mood. It is a 21 item self-report questionnaire of symptoms typically associated with depression. For instance, participants were asked to identify the extent to which they feel sad, discouraged about the future, or cry more than usual. All outcome scores except for depression were scaled to t-scores according to representative age and sex distributions, with the mean expected score of 50 and a standard deviation of 10. Similar to the memory tests, the Digit Symbol Substitution Test was not administered in years five and six due to a strong practice effect. Such practice effects are common in longitudinal studies that involve repetitive testing and the best correction in this study was to take a break in these tests. After two years of collecting only exposure and mood data, permitting a break from the cognitive measures, we returned to administering the memory measure again in years seven and eight. We piloted the Digit Symbol Substitution Test late in year six and it still showed a strong practice effect, so we deleted it from the cognitive battery.

The analysis used raw scores from the BDI, which has a possible range of 0–63, with higher scores indicating higher levels of Depression. Additionally, the analysis of Depression used clinically relevant cutoffs to categorize participants into the following categories: minimally depressed (BDI score 0–13), mildly depressed (14–19), moderately depressed (20–28), and severely depressed (29–63).

Generalized estimating equations (GEE) accounted for within-person correlations of data. In the bivariate analysis, razor clam consumption groups were compared on baseline characteristics, using the first available data for the baseline characteristics, but using year-specific consumption data for each participant.

The multivariable analysis utilized similar GEE models to analyze differences in cognitive outcomes between different groups but adjusted for the confounding effect of the year of study. One year-specific predictor variable (razor clam consumption group, gender, marital status, education level, employment, alcohol use, or marijuana use) was allowed in each model, plus a categorical variable for the year of study. Only these two independent variables (primary predictor plus year of study) were entered into each model, allowing for valid comparisons of results between models for different predictors and outcomes. The adjustment for the year of study in particular allowed for valid results using multiple years’ worth of data, accounting for the fact that scores and instruments fluctuated between years.

## 3. Results

The sample included 500 adult participants, aged 18–64, who had data for 1873 visits. Table 1 reports the sample’s characteristics, using the first available data for each participant. Majorities of the sample were female (58%) and not currently married (70%). More than 50% of the participants had a high school education or less, and 56% reported current employment. Few participants indicated that they consumed alcohol more than once per week (8%) or that they had used marijuana within the past year (11%).

At approximately 33% of visits, the participant indicated that they had not eaten razor clams within the past year, and 22% of participants indicated that they were high consumers (15+ clams eaten per month). Non-consumers tended to be younger than consumers. The three consumer groups had similar characteristics on marital status, education, employment, and marijuana use.

In the first year, the total sample scored within the average range on short delay recall, long delay recall, and Digit Symbol measures (Table 2). The mean score on the Beck’ Depression Inventory (BDI) was 10.1 in year 1, with 75% of respondents showing minimal signs of depression.

A series of multivariable GEE models (Table 3) accounted for within-person correlation and the confounding effect of the year of study (and therefore, the confounding effect of changing instruments between years). Of the five tested outcomes, razor clam consumption was most significantly associated with short-term recall and total recall, with high consumers scoring approximately one point lower on this scale than no- and low-consumers. High consumers did not have significantly lower scores than no- and low-consumers on long delay recall, Digit Symbol, or Depression. Higher educated participants had higher mean scores on all outcomes except for Depression, for which they scored lower. Those reporting current employment also scored higher on all outcomes except for Depression. The only difference between men and women was that men ate more razor clams and were more heavily represented in the high consumer group. There were no differences in memory outcome by age, most likely due to the fact that there were few study participants over the age of 60 in this sample.

## 4. Discussion

Overall, exposure to DA via dietary consumption of 15 or more razor clams/month over eight years resulted in a slight decline in total recall, i.e., a memory summary score. This finding was maintained after taking into consideration demographic factors (age, sex), study year, and repeated and modified measures. These high consumers scored approximately one point lower on the total recall scale than no- and low-consumers. Long-delayed recall, depression, and clerical speed and accuracy remained stable over the same time period. Findings are similar to those previously reported by Grattan et al. [3] whereby consuming more than 15 razor clams per month (high consumers) was associated with a mild memory decline within the context of otherwise normal, stable cognition and mood. Similar to the current study the impact was low. In both studies, despite the slight decrement, the overall memory scores remained within the average range compared to referenced norms. This minimal memory decline may reflect the relatively low levels of DA in the harvested razor clams, a negligible effect of repeated exposures and/or the lack of a cumulative effect of the toxin over time. Alternatively, it is possible that memory effects (if any) after the initial dietary exposure in this study were reversible. In contrast, the acute cases of ASP associated with much higher DA level exposures during the Montreal outbreak were permanent. Further support for reversibility may be found in a study by LeFebvre and colleagues [28] using a mouse model. These authors repetitively exposed the rodents to DA which led to spatial learning deficits. Once the exposures were discontinued, full recovery of spatial learning occurred.

Another relevant consideration is whether the statistically significant “mild” memory decline is clinically or functionally meaningful. Similar to the four-year outcome study [3], the memory decline identified in this study was minimal and most likely of no clinical significance to the NA population under study. With respect to functional significance, studies of “Everyday Memory” (EM) in a subgroup of this population were previously conducted [1]. Findings indicated an association between problems with EM and consumption of razor clams with putatively safe levels of DA (<20 ppm) within two to three weeks after a large community clam harvest. Participants who continued to consume 15 or more razor clams per month over the following year were also more likely to report problems with everyday memory than others.

The current study has several limitations. The first was reliance on self-reported consumption data and average DA levels at source beaches to estimate exposure. Despite our best efforts [29], there is currently no known diagnostic biomarker for identifying DA exposure. Such a biomarker would be essential for specifically identifying the extent of human exposure and its relationship to memory outcomes. Second, annual assessments may be too infrequent to detect an immediate impact of the exposure and recovery, if any. More frequent, post-meal assessments would be indicated to manage this limitation. Another limitation is that there were too few older participants in the study to determine if they are more vulnerable to the memory effects of DA exposure than younger adults. Studies focusing on the effects of DA on the aging NA population are indicated. Finally, the generalizability of these findings to other non-NA groups remains uncertain. The nature and extent of impacts to non-NA groups will be largely related to whether or not they have access to and consume a lot of razor clams. Future studies with a broader community sample are indicated.

DA is a potentially significant public health problem in coastal communities where toxic *Pseudo-nitzschia* is at risk of proliferation. Current regulatory guidance has been protective against acute high-level exposures and ASP. The potential impacts of repetitive, lower levels exposure are, however, are of potentially broader concern. Public health outreach in the US Pacific NW with Tribal and community leaders; fish and wildlife managers; state regulatory agencies; and health care professionals is ongoing. This includes recommendations that (1) adults avoid consuming more than 15 razor clams/month over the course of a year using any method of preparation and (2) advisories to vulnerable groups such as pregnant women, nursing mothers, and the elderly. Risks remain to other people in coastal communities where DA is endemic. Collaboration between HAB managers and local health and fisheries department is essential for communicating these recommendations and devising additional preventive education, interventions or other solutions to protect brain health.

## 5. Conclusions

The findings of this study suggest that high consumption of razor clams with putatively safe levels of DA over time may result in a mild memory decrement within the context of otherwise stable cognitive abilities. This is likely not of clinical significance, but within the context of previous studies, suggests that everyday memory or functional status may be disrupted. Considerations of reversibility of the memory difficulties associated with low-level exposures are raised as well as the possible impact on cognitive decline aging NAs. Finally, there is reason to believe that there may be a continuum of memory problems associated with varying levels of DA exposure. Thus, the unitary construct of ASP in relationship with DA may need to be modified to include a broader range of DA neurotoxicity.

## Figures and Tables

**Table 1:** Baseline characteristics and razor clam consumption.

	Participant’s First Visit (n = 500)	All Data All Visits (n = 1873 Visits)			
		Razor Clam Consumption			
	Total	None	Low	High	*p* ^p^
			(1–14 Clams/Month)	(15+ Clams/Month)	
**Number (row %)**	500	607 (32%)	861 (46%)	405 (22%)	
**mean (SD)**					
**Age at enrollment**	38.9 (12.7)	38.3 (12.9)	41.9 (12.2)	43.0 (11.0)	0.01
	**n, column %**				
	**(n = 500 participants)**				
**Gender**					0.15
Male	210 (42%)	29%	46%	24%	
Female	290 (58%)	35%	46%	20%	
**Marital status**					0.64
Not married	331 (70%)	32%	45%	23%	
Currently married	144 (30%)	32%	47%	21%	
**Education level**					0.18
Less than high school	118 (24%)	29%	42%	29%	
High school graduate	189 (38%)	37%	42%	21%	
College 1–3 years	162 (33%)	30%	52%	18%	
College 4+ years	29 (6%)	25%	54%	21%	
**Alcohol use**					0.05
Weekly or less often	433 (92%)	32%	46%	21%	
More often than weekly	36 (8%)	27%	44%	29%	
**Marijuana use**					0.48
No, past year	404 (89%)	34%	46%	21%	
Yes, past year	49 (11%)	32%	41%	27%	
**Year of study**					<0.01
Year 1	485	21%	51%	28%	
Year 2	393	33%	51%	17%	
Year 3	340	39%	44%	18%	
Year 4	314	40%	36%	23%	
Year 7	178	37%	43%	20%	
Year 8	163	35%	43%	22%	

^p^ Generalized estimating equations, using data from all years for all participants, accounting for within-participant clustering.

**Table 2:** Distribution of cognitive outcomes, by year of study (n = 500).

	Year of Study						*p*-Value ^p^
Measure	1	2	3	4	7	8	
**CVLT-II or HVLT, ^c^ mean t-score (SD)**							
Total Recall	37.8 (10.3)	42.5 (10.5)	46.9 (11.6)	47.2 (12.2)	31.2 (10.3)	33.4 (10.3)	<0.01
Short Delay Recall	40.9 (9.1)	43.9 (9.3)	47.4 (10.0)	47.9 (10.6)	N/A	N/A	<0.01
Long Delay Recall	41.0 (9.4)	44.4 (9.7)	46.6 (10.4)	47.5 (10.9)	33.9 (12.2)	34.5 (12.0)	<0.01
**WAIS-III Digit Symbol, mean t-score (SD)**	45.2 (9.2)	47.3 (9.1)	47.8 (9.4)	47.8 (9.6)	N/A	N/A	<0.01
**Beck’s Depression Inventory**							
Mean raw score, SD	10.1 (9.1)	10.5 (9.8)	9.9 (9.4)	9.5 (9.7)	6.0 (7.4)	6.9 (7.2)	<0.01
Minimally depressed, %	75%	71%	73%	76%	88%	82%	<0.01
Mildly depressed, %	11%	12%	13%	10%	5%	10%	
Moderately depressed, %	8%	10%	8%	9%	6%	7%	
Severely depressed, %	6%	7%	6%	6%	2%	1%	

CVLT: California Verbal Learning Test. HVLT: Hopkins Verbal Learning Test. WAIS: Wechsler Adult Intelligence Scale. SD: standard deviation; ^c^ CVLT-II was administered in Years 1–4. HVLT was administered in Years 7–8. ^p^ Generalized estimating equation, with year as a categorical predictor and the outcome as continuous. Short Delay Recall and WAIS Digit Symbol were not administered in Years 7–8.

**Table 3:** Year-adjusted relative expected cognitive scores, by baseline characteristics, over time.

	CVLT-II/HVLT						WAIS-II		BDI	
	Total Recall		Short Delay				Digit Symbol			
Predictor	Coef. (95% CI)	*p*	Coef. (95% CI)	*p*	Coef. (95% CI)	*p*	Coef. (95% CI)	*p*	Coef. (95% CI)	*p*
**Razor clam consumption**										
None/Low (0–14 clams/mo.)		0.03		0.04		0.28		0.13		0.45
High (15+ clams/mo.)	−1.0 (−2.0, −0.1)		−1.1 (−2.1, −0.0)		−0.6 (−1.6, +0.5)		−0.6 (−1.3, +0.2)		−0.3 (−1.2, +0.5)	
**Gender**										
Male	+0.1 (−1.6, +1.8)								+3.1 (+1.8, +4.5)	
Female		0.94	−1.6 (−3.1, −0.1)	0.04	−0.4 (−2.1, +1.2)	0.59	+6.1 (+4.6, +7.6)	<0.01		<0.01
**Marital status**										
Not married			+1.2 (−0.4, +2.8)		+2.0 (+0.2, +3.7)		+3.7 (+2.0, +5.3)		−0.3 (−1.8, +1.2)	
Currently married	+1.8 (−0.0, +3.7)	0.05		0.15		0.03		<0.01		0.68
**Education level**										
High school or less	+5.5 (+3.9, +7.2)						+5.8 (+4.3, +7.3)			
At least some college		<0.01	+3.2 (+1.7, +4.7)	<0.01	+4.9 (+3.3, +6.5)	<0.01		<0.01	−1.9 (−3.3, −0.5)	0.01
**Employment**										
Not working	+1.9 (−1.2, +4.9)		+2.1 (−0.6, +4.8)		+1.6 (−1.3, +4.6)		+0.3 (−2.4, +3.0)		−3.8 (−6.3, −1.3)	
Working, Manual labor	+6.3 (+4.4, +8.2)	0.22	+4.0 (+2.3, +5.7)	0.13	+5.2 (+3.3, +7.0)	0.27	+6.6 (+4.9, +8.3)	0.83	−2.2 (−3.8, −0.7)	<0.01
Working, Office work	+4.0 (+1.7, +6.4)	<0.01	+2.3 (+0.2, +4.4)	<0.01	+2.2 (−0.0, +4.4)	<0.01	+2.5 (+0.4, +4.6)	<0.01	−1.1 (−3.1, +0.8)	0.01
Working, unspecified		<0.01		0.03		0.05		0.02		0.25

CVLT: California Verbal Learning Test. HVLT: Hopkins Verbal Learning Test. WAIS: Wechsler Adult Intelligence Scale. BDI: Beck Depression Inventory. CI: confidence interval. T-scores were used for all outcomes except for BDI, which used raw scores. Confidence intervals and p-values from generalized estimating equations (GEE), adjusted for year of study. A separate GEE model was built, adjusted for year of study, for each combination of independent variable and outcome (e.g., this table includes seven predictor variables and five outcome variables, meaning that 35 total GEE models are represented, each of which has two independent variables: the predictor variable listed, plus year of study). Negative value indicates a group that had a lower expected score than the reference group.

## Data Availability

Data supporting this results can be provided from the Principal Investigator of this study (LMG) to qualified scientists upon request to the extent that full confidentiality of participating tribes and tribe members is maintained. At the request of the participating tribal communities, there are no publicly archived data sets associated with this study.
